# 3D Fractals as SERS Active Platforms: Preparation and Evaluation for Gas Phase Detection of G-Nerve Agents

**DOI:** 10.3390/mi9020060

**Published:** 2018-01-31

**Authors:** Marta Lafuente, Erwin J. W. Berenschot, Roald M. Tiggelaar, Reyes Mallada, Niels R. Tas, Maria P. Pina

**Affiliations:** 1 Nanoscience Institute of Aragon, Department of Chemical & Environmental Engineering, University of Zaragoza, Edif I+D+i, Campus Río Ebro, C/Mariano Esquillor, s/n, 50018 Zaragoza, Spain; martalaf@unizar.es (M.L.); rmallada@unizar.es (R.M.); 2 Mesoscale Chemical Systems, MESA+ Institute for Nanotechnology, University of Twente, P.O. Box 217, 7500 AE Enschede, The Netherlands; j.w.berenschot@utwente.nl (E.J.W.B.); r.m.tiggelaar@utwente.nl (R.M.T.); 3 MESA+ NanoLab cleanroom, MESA+ Institute for Nanotechnology, University of Twente, P.O. Box 217, 7500 AE Enschede, The Netherlands; 4 Networking Research Center of Bioengineering, Biomaterials, and Nanomedicine, CIBER-BBN, 28029 Madrid, Spain

**Keywords:** SERS, 3D-fractal structures, corner lithography, Au@citrate, gas sensing, nerve agents, ppm detection

## Abstract

One of the main limitations of the technique surface-enhanced Raman scattering (SERS) for chemical detection relies on the homogeneity, reproducibility and reusability of the substrates. In this work, SERS active platforms based on 3D-fractal microstructures is developed by combining corner lithography and anisotropic wet etching of silicon, to extend the SERS-active area into 3D, with electrostatically driven Au@citrate nanoparticles (NPs) assembly, to ensure homogeneous coating of SERS active NPs over the entire microstructured platforms. Strong SERS intensities are achieved using 3D-fractal structures compared to 2D-planar structures; leading to SERS enhancement factors for R6G superior than those merely predicted by the enlarged area effect. The SERS performance of Au monolayer-over-mirror configuration is demonstrated for the label-free real-time gas phase detection of 1.2 ppmV of dimethyl methylphosphonate (DMMP), a common surrogate of G-nerve agents. Thanks to the hot spot accumulation on the corners and tips of the 3D-fractal microstructures, the main vibrational modes of DMMP are clearly identified underlying the spectral selectivity of the SERS technique. The Raman acquisition conditions for SERS detection in gas phase have to be carefully chosen to avoid photo-thermal effects on the irradiated area.

## 1. Introduction

Surface-enhanced Raman scattering (SERS) spectroscopy is based on the enormous enhancement of Raman scattering of molecules adsorbed on suitable metallic nanostructures. The amplification of signals in SERS only occurs in very close vicinity (ca. < 10 nm) to the metal substrate and relies on the electromagnetic interaction of light with metals. There are two widely accepted mechanisms for SERS, i.e., the chemical mechanism (CM) and the electromagnetic mechanism (EM). CM is based on a charge transfer between the molecule and the substrate. Because of this transfer, the polarizability of the molecule increases and consequently, the Raman cross-section of the molecule. This enhancement is usually 10–10^2^. However, the EM is based on the enhancement of the local electromagnetic field that results in a significant increase in Raman signal, proportional to |E|^4^. This increase can become 10^8^ or more. The interaction of the light with the metal substrate produces large amplifications of the laser field through excitations generally known as plasmon resonances [1]; either localized surface plasmon resonances (LSPRs) for 0D and 1D nanostructures or propagating surface plasmon polaritons (SPP) for 2D tunable nanostructures [2]. Nowadays, Surface Enhanced Raman Scattering (SERS) outstands as one of the leading techniques for label-free ultrasensitive vibrational fingerprinting of a variety of molecular compounds [3,4,5,6,7].

In a recent review paper on explosives and chemical threats detection [8], SERS has been identified as key technique because it combines several attractive features such as ultrasensitivity, high speed, simple sample manipulation, comparatively low cost, multiplexing detection capability (narrow bands of vibrational Raman scattering) and portability. Despite its exceptional advantages, additional efforts on the fabrication of SERS substrates are still required to circumvent the following limitations: deficient target-substrate contact, poor homogeneity and reproducibility of the signal within a substrate and limited re-usability of the substrates. In particular, the degree of control and reproducibility of commercial as well as research-based SERS substrates are still questionable and significant improvements in terms of performance, process standardization and sample-to-sample reproducibility need to be done.

The development of robust, reproducible and cost-effective SERS substrates containing a large number of hot spots that can give rise to high enhancement factors; and capable to co-localize the targeted molecules in the hot spots is therefore a key requirement towards the widespread use of SERS as practical analytical technique [9]. Most of the reported SERS substrates take advantage of the hot spots limited to a single cartesian plane. Thus, the scattered volume calculated from the laser spot and the laser-probe interaction depth is widely infra-utilized. Accordingly, 3D SERS active substrates with considerable extension in the z-direction are becoming an active focus of research [9,10,11,12,13,14]. Hsu et al. presented a Sierspinki carpet fractal structure by a solid-state electrochemical patterning technique on 40 nm thick Ag film [15] as 3D platform for SERS EM field enhancement. The fractal structure includes features of sizes ranging from 150 nm to 4 μm. The structures were protected with an Al_2_O_3_ layer of l.6 nm to isolate the CM effect. The highest electromagnetic enhancement was observed for the size-range 150/750 nm, but also the intensity of the photon counts increased for the 1.2 μm and even for 4 μm structures, by a factor of 100 and 16, respectively. The EM field supported by the fractals further extends spatially compared to continuous 2D silver surfaces.

The combination of self-assembly of NPs with top-down fabrication of periodic surfaces has demonstrated clearly benefits in terms of large-scale and highly reproducible preparation [8,9,10]. Theoretically, the number of hot spots in the 3D nanostructures is greater than that of the 2D counterparts. Furthermore, the extension of a SERS substrate from 2D to 3D leads to about a larger overall surface area, and this promotes adsorption and detection of more target molecules. Finally, the tolerance in focus (mis)alignment along the z-direction could lead to more reproducible SERS signals.

This work explores the use of 3D-fractals with metallic coatings as SERS active substrates for label free detection of G-nerve agents in gas phase. It is well known that Chemical Warfare Agents (CWAs) are poor Raman scatterers with cross-sections in the range of 10^−29^ cm^2^ sr^−1^ molecule^−1^. This characteristic feature precludes any possibility of analyte detection at low concentration levels without special enhancement processes. Available detection and identification systems for airborne chemical threats remain sorely lacking in terms of the sensitivity and selectivity achievable in the short response time that a real incident would require. CWAs are especially insidious in this respect, given the low concentrations needed to cause potentially lethal effects (for instance, with Sarin gas the value stands at 0.064 ppm for 10 mins) [16]. Indeed, field experiences in simulated scenarios reveal numerous false alarms and error rates so high that it becomes nearly impossible to identify in real time unknown or not explicitly searched-for agents.

The goals of this paper are to characterize the Raman signal intensity enhancements of these 3D-fractal structures as a function of generation of bifurcations (denoted as 1G and 3G fractals) and their performance is compared with non-patterned colloidal gold films. Moreover, the applicability of 3D-fractal microstructures for the spectral identification of Sarin surrogate, i.e., dimethyl methylphosphonate, in gas phase at ppm concentration level is demonstrated.

## 2. Experimental Section

### 2.1. 3D-Fractal Fabrication

**Figure 1:**
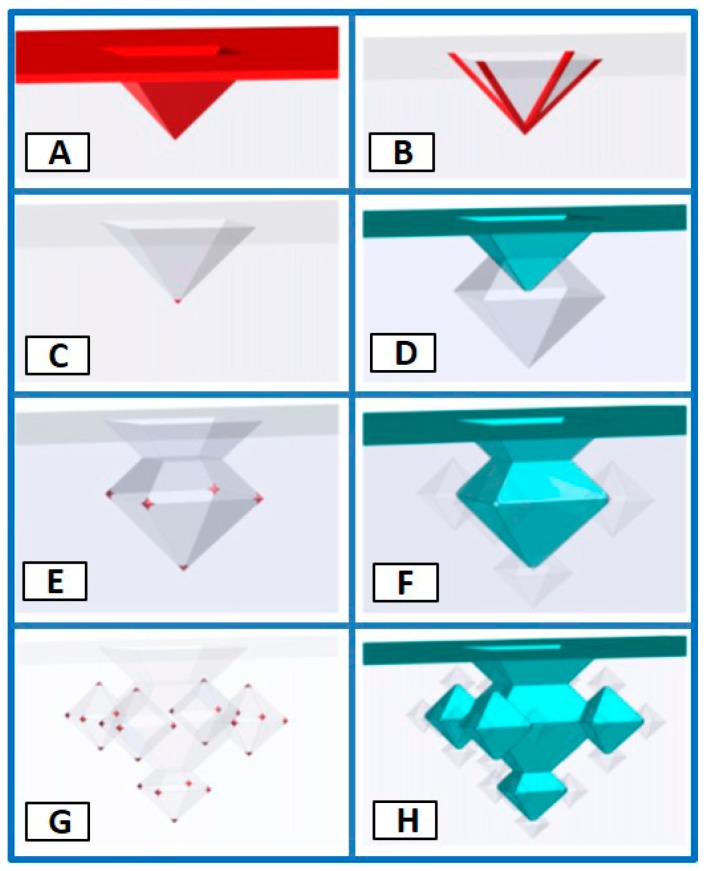
Octahedral 3D-fractal fabrication scheme, showing in (**A**–**D**) the evolution of the first generation (1G), in (**E**,**F**) the fabrication of the second generation (2G) and in (**G**,**H**) the realization of the third generation (3G) fractals (adapted from [17]).

The method of engineering of a 3D-fractal structure is based on a combination of anisotropic etching of silicon and corner lithography [17,18]. Basically, a silicon (Si) wafer with thermally grown SiO_2_ was patterned in buffered hydrofluoric acid (BHF) using a resist mask with a regular pattern of holes (25 μm diameter and 25 μm periodicity). The silicon which was unprotected, was anisotropically etched in potassium hydroxide (KOH) in order to create inverted pyramidal-shaped pits. The remaining oxide mask was stripped. Next, the wafer was uniformly coated with 160 nm of low-pressure chemical vapor deposited silicon nitride (Si_3_N_x_) (see Figure 1A). The next step, called corner lithography, was used to isotropically etch the silicon nitride in hot phosphoric acid (H_3_PO_4_) (see Figure 1B) and leaves only a dot in the corner of the inverted pyramid (see Figure 1C). The following step was a LOCOS process (LOCal Oxidation of Silicon). In this stage, bare silicon was locally oxidized at 1100 °C (45 mins, yielding 77 nm SiO_2_) using the silicon nitride dots as mask. The silicon nitride in the corner of each inverted pyramid was stripped with H_3_PO_4_. Next, the unprotected silicon in the pyramidal apex was etched anisotropically using tetra methyl ammonium hydroxide (TMAH; 125 mins) which formed a single octahedral shape feature at the vertex of the pyramid (see Figure 1D), denoted as 1 G (1st generation 3D-fractal). The first level of processing was finished by stripping of SiO_2_ and depositing around 88 nm of nitride. The entire process was repeated in order to create the second (2G; see Figure 1F), and third generation (3G; see Figure 1H) of fractal microstructures. To facilitate handling of the hollow 3D fractal surfaces, anodic bonding of the processed silicon wafer to a Mempax glass wafer (500 μm thick) was performed, followed by dissolvation (back-etching) of the silicon. Thus, all 1G and 3G silicon nitride fractals are positioned on a glass substrate.

### 2.2. Preparation of 3D-Fractals Active SERS Substrates

**Figure 2:**
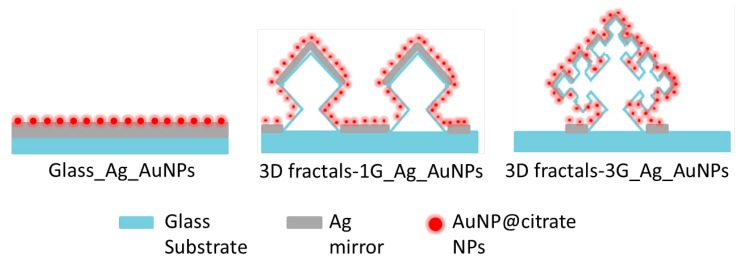
Illustrations of reference (**left side**), 1G fractal (**middle**) and 3G fractal (**right side**) surface-enhanced Raman scattering (SERS) active substrates studied in this work.

The hierarchical 3D-fractal platforms are fabricated by combining corner lithography and anisotropic Si-etching with electrostatically driven Au NPs assembly. The top-down fabrication sequence creates microstructured platforms required to extend the SERS-active area into 3D, and the self-assembly of Au NPs ensures homogeneous coating of SERS active Au NPs over the entire microstructured platforms (see Figure 2). Furthermore the fractals could extend the EM field enhancement.

Spherical gold nanoparticles 22 nm in size, Au@citrate NP, were synthesized via a modified version of the Turkevich-Frens method [19]. Briefly, 50 mL of aqueous solution (1.1 mM) of HAuCl_4_ (50% Au basis) was heated to 70 °C under stirring, and then 5 mL of preheated sodium citrate solution (3.8 mM) was added. The solution was kept at 70 °C until a red-wine colour appeared, circa 10 mins. Then, the liquid was allowed to cool to room temperature. The synthesis experiments have been performed by the platform of Production of Biomaterials and Nanoparticles of the NANBIOSIS ICTS, more specifically by the Nanoparticle Synthesis Unit of the CIBER in BioEngineering, Biomaterials & Nanomedicine (CIBER-BBN).

Following the fabrication of 3D fractals, they were metallized with silver (99.99% silver pellets from Kurt J. Lesker Company, Jefferson Hills, PA, USA) via electron beam evaporation (Edwards auto-500, 3∙10^−7^ mbar, 32 mA, 5.3 KV). In a second step, gold nanoparticles (Au@citrate NPs) were assembled on the substrates by electrostatic interactions to generate a 3D monolayer-over-mirror configuration. For such purposes, the SERS substrates were incubated in a poly(diallyl-dimethilammonium) chloride aqueous solution (PDDA), 0.2% wt for 4 h; followed by rinsing with deionized water. Afterwards, the substrates were immersed in Au@citrate nanoparticles solution (0.19 mg/mL) for 16 h at 4 °C. Then the SERS substrates were rinsed with deionized water and dried at room temperature. Table 1 shows the main characteristics of the SERS active substrates studied in this work, where reference samples used for comparison purposes are also included.

### 2.3. SERS-Raman Measurements

An alpha300 R- confocal Raman Imaging® spectrometer of WITec (Wissenschaftliche Instrumente und Technologie GmbH, Ulm, Germany) was used (480 nm as lateral spatial resolution). Raman-SERS spectra were collected in backscattering geometry. Excitation of the samples was carried out with laser 785 nm at room temperature. Although this wavelength does not correspond to the maximum absorption of the SERS substrates (see Figure S1 of Supplementary Materials), this laser has been selected to avoid the photodegradation of the dimethyl methylphosphonate (DMMP) molecule that we observe when 633 nm laser was used.

In this work, rhodamine 6G (R6G) was chosen as probe molecule and the characteristic band for C-C stretching exhibited at 1512 cm^−1^ was selected for Enhancement Factor (EF) and SERS Gain quantification.

This EF quantifies how much the Raman signal is amplified with respect to normal conditions, giving information on the field enhancement provided by the structures. It is calculated to assess the SERS activity of the prepared substrates and it is given by:

(1) $$\mathrm{EF}=\frac{\frac{I_{\mathrm{SERS}}}{N_{\mathrm{SERS}}}}{\frac{I_{\mathrm{Raman}}}{N_{\mathrm{Raman}}}}$$

where I_SERS_ is the Raman-band intensity corresponding to the number of molecules analyzed on the SERS substrate, N_SERS_, and I_Raman_ and N_Raman_ are the intensity and number of molecules without the presence of the SERS substrate, respectively.

To calculate N_Raman_ a Raman spectrum was acquired on a liquid droplet of R6G 1 mM aqueous solution at 50×, 15 mW, 100 s acquisition conditions: The number of R6G molecules within the interaction volume of the laser, 2.1 × 10^−12^ cm^3^, was calculated as follows [20].

(2) $$N_{\mathrm{Raman}}=6.023\times 10^{23}\frac{\mathrm{molecules}}{\mathrm{mol}}\times \left(R6\mathrm{Gconcentration}\right)\times \left(\mathrm{interaction}\;\mathrm{volume}\right)$$

To establish the values for I_SERS_ a 2 μL droplet of 1 μM R6G (aqueous solution) was deposited on the different SERS substrates (see Table 1). The droplet was allowed to evaporate. The area-size covered by the dried droplet on the different substrates was measured and the total 3D surface area, including the fractal structures, was calculated. It is assumed that the R6G molecules are distributed evenly across the dried spot. The spectra were recorded for R6G at 20×, 1.6 mW, 0.1 s acquisition conditions. The R6G spectrum of the SERS substrate was measured in the center of the droplet once dried. The number of R6G molecules within the effective laser spot, N_SERS_ was estimated by the following equation [21]:

(3) $$N_{\mathrm{SERS}\;}=\;\frac{\left(R6G\;\mathrm{molecules}\;\mathrm{in}\;\mathrm{the}\;\mathrm{droplet}\right)\times \left(\mathrm{irradiated}\;\mathrm{area}\right)}{\mathrm{surface}\;\mathrm{area}\;\mathrm{of}\;\mathrm{the}\;\mathrm{dried}\;\mathrm{droplet}}$$

On the other hand, the SERS Gain provides quantitative information on the signal gain that one has to expect from a specific SERS sensor with respect to a reference Raman experiment. The SERS Gain is calculated as the ratio between the SERS and Raman Intensities, I_SERS_ and I_Raman_ respectively, normalized to the different powers (P_SERS_ and P_RAMAN_), integration times (t_SERS_; t_Raman_) and molecular concentration (c_SERS_; c_Raman_) used in the experiment. Therefore, the SERS Gain was calculated by the following equation [22]:

(4) $$\mathrm{SERS}\;\mathrm{Gain}=\;\frac{I_{\mathrm{SERS}}/\left(t_{\mathrm{SERS}}\times P_{\mathrm{SERS}}\times c_{\mathrm{SERS}}\right)}{I_{\mathrm{Raman}}/\left(t_{\mathrm{Raman}}\times P_{\mathrm{Raman}}\times c_{\mathrm{Raman}}\right)}$$

**Figure 3:**
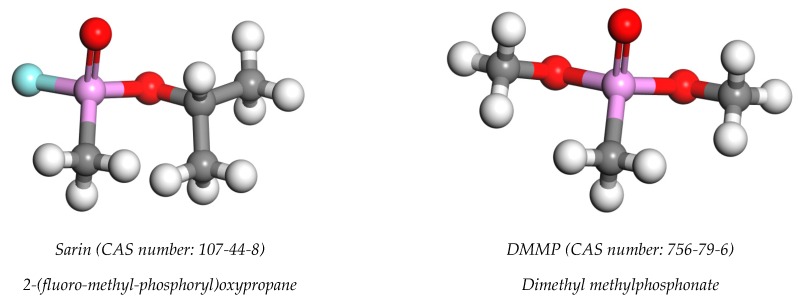
Structural analogies of Sarin and dimethyl methylphosphonate (DMMP) surrogate. Color legend: P, purple; F, blue; C, grey; O, red; and H, white.

**Figure 4:**
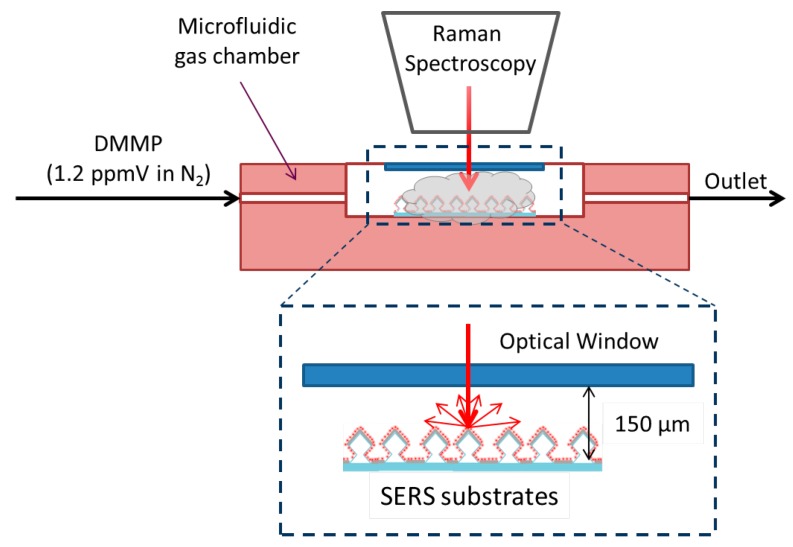
Experimental set-up for continuous SERS measurements of DMMP in gas phase.

SERS experiments for detection of traces of CWA vapors were carried out with DMMP, often used as a Sarin gas simulant thanks to its chemical structure similarities (Figure 3) and its much lower toxicity. For measuring DMMP in gas phase, the SERS substrate was mounted on a home-made gas chamber (2.7 × 10^−2^ cm^3^) placed at an angle of 90° with respect to the 785 nm laser excitation beam (Figure 4). A nitrogen stream (10 STP cm^3^ min^−1^) was passed through a bubbler containing liquid DMMP, placed in a thermal bath at room temperature, and fed to the microfluidic chamber. The SERS measurements were immediately performed without any stabilization period. The value of the concentration of DMMP in the feed stream was calibrated with a permeation tube (MT-PD-Experimental, 107-100-7845-HE3-C50) of known concentration and corresponds to 1.2 ppmV. The 785 nm excitation laser was coupled through 20× objective (Numerical Aperture, N.A., 0.5; spot diameter 1.9 µm) or 50× objective (N.A., 0.8; spot diameter 1.2 µm) respectively. The SERS spectra are shown after baseline subtraction using WITec Control 1.60.

### 2.4. Characterization Techniques

Scanning electron microscopy (SEM) images were obtained using a FEI INSPECT 50 system equipped with a FEG source of electrons. ImageJ analysis was used to obtain the Au@citrate NP density onto the SERS substrates from three different SEM images, randomly selected of each one. Atomic force microscopy (AFM) measurements (Multimode 8 from Veeco-Bruker; tip, OMCL-AC240TN-W2 from OLYMPUS, XY resolution < 20 nm) were conducted in tapping mode in air to investigate the topography of the metallic coatings. Roughness was estimated by Gwyddion 2.45 analysis of topography images. The UV measurements were performed in a Jasco V-670 spectrometer equipped with a DRIFT chamber for the measurement of solid surfaces.

## 3. Results and Discussion

### 3.1. Morphological Characterization of the 3D Fractals SERS Active Substrates

**Figure 5:**
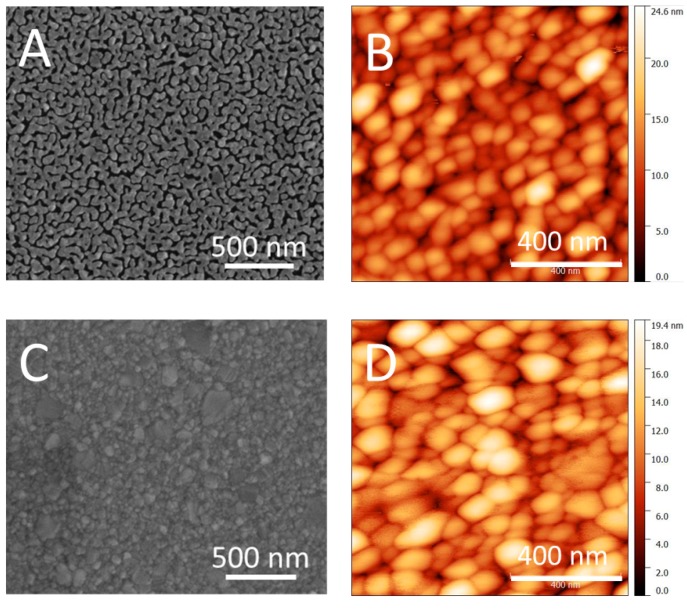
SEM and topological AFM images of SiO_2_ surfaces upon metallization with 12 nm (**A**) and (**B**) and 77 nm (**C**) and (**D**) of Ag, respectively.

Recently, plasmonic systems consisting of metal nanoparticles separated from metal films by nanometer scale gaps have attracted great attention as SERS substrates, because the LSPRs of metal nanoparticles can couple with the propagating SPPs at the surface of metal films when precise gap regions between metal nanoparticles and the films are provided [23]. In this work, a preliminary evaluation of the minimal thickness for the silver coating was performed on flat, oxidized silicon wafers (Siltronix Double side polished Si (100) with ca. 1 µm of SiO_2_) previous to metallization of 3D-fractal structures. Two different silver evaporation times were selected, i.e., 90 and 500 s, which result in film thicknesses, measured by AFM, of 12 ± 1 and 77 ± 3 nm respectively. As we can observe in Figure 5, when the amount of silver deposits increases, the formation of a continuous layer is enabled. In addition, the average roughness decreases slightly with increasing film thickness, i.e., 2.8 ± 0.1 nm and 2.6 ± 0.2 nm. Accordingly, 3D-fractal structures were metallized with 77 nm thickness of silver.

**Figure 6:**
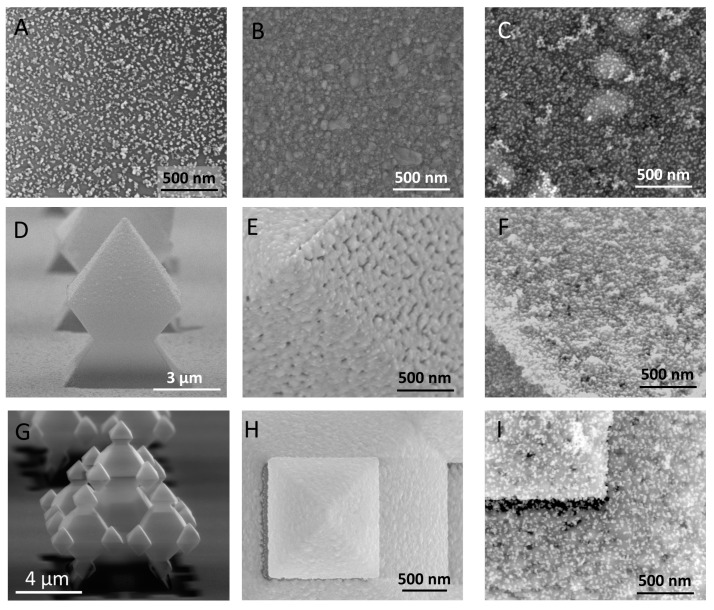
SEM characterization of the SERS substrates herein studied. (**A**) Glass_AuNPs, (**B**) Glass_Ag, (**C**) Glass_Ag_AuNPs, (**D**) Bare 1G fractal, (**E**) Silver-coated 1G fractal (1G_Ag) (**F**) SERS active 1G fractal (1G_Ag_AuNPs), (**G**) Bare 3G fractal, (**H**) Silver-coated 3G fractal (3G_Ag), (**I**) SERS active 3G fractal (3G_Ag_AuNPs).

The assembly of Au@citrate NPs was performed on the different SERS substrates using the electrostatic interaction between the negatively charged sodium citrate groups anchored on the Au@citrate NPs and the positively charged PDDA layer on the SERS substrate. Figure 6 shows the results of Au@citrate NPs deposition over 2D and 3D active SERS substrates. In general, the experimental procedure yielded a homogeneous monolayer of Au@citrate NPs with a surface density above 600 NPs/µm^2^, i.e., a coverage degree of around 25%. As it was expected, no differences are observed for samples without Ag due to the effectiveness of the intermediate PDDA coating. The Au@citrate NPs density on the flat surfaces is around a 6% higher compared to the 3D-fractal surfaces. These minor deviations are attributed to the higher surface roughness for the Ag coatings when evaporated on flat substrates compared to fractals (see Figure 6B,E,H).

### 3.2. SERS Performance for Au Monolayer over Mirror Configuration

**Figure 7:**
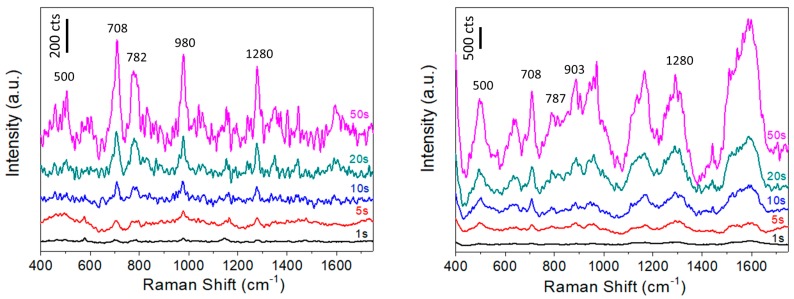
SERS response of Glass_Ag_AuNPs (**left**) and Glass_AuNPs (**right**) samples upon exposure to DMMP vapor (1.2 ppmV) as a function of integration time. Acquisition conditions: 50×, 5 mW.

The SERS performance of the Au monolayer-over-mirror configuration, i.e., of the Glass_Ag_AuNPs sample, is highlighted by comparing it with Au@citrate NPs coated onto non-metallized glass substrates, i.e., the Glass_AuNPs sample. Figure 7 shows the SERS response of both samples under identical conditions upon exposure to DMMP vapor, at different integration times. On the silver coated sample, i.e., Glass_Ag_AuNPs, the characteristic molecular fingerprint of DMMP molecules adsorbed on the SERS substrate is clearly distinguished [24]. As the integration time increases, more signal is collected by the detector, and for an integration time of 20 s (or larger) is possible to obtain a signal with a value of around 200 cts for the more intense peak at 710 cm^−1^. As was explained in the introduction section, due to the toxicity of Sarin gas a fast detection is pursued. The main vibrational modes of DMMP: 710 cm^−1^ (ν (P-CH_3_)), 782 cm^−1^ (ν_as_ (O-P-O)), 980 cm^−1^ and 1280 cm^−1^ (ν (P = O) can be perfectly identified when using Au monolayer-over-mirror configuration. On the other hand, the non-metallized Glass_AuNPs sample gives a higher SERS intensity but the spectral fingerprint of the target is hindered by the Raman lines for citrate coating, i.e., 887 cm^−1^–950 cm^−1^ (ν (C-COO)) and 1167 cm^−1^ (δ (COO)). In addition, a broad band in the region 1450 to 1700 cm^−1^, attributed to amorphous carbon, is observed. The carbon formation agrees with the photodecomposition of the citrate molecules covering the Au NPs. Our hypothesis is that in the case of a non-metallized surface, the local temperature on the substrate increases up to levels that lead to photodecomposition due to the poor thermal conductivity of the underlying glass (1 W/K·m) compared to Ag films (410 W/K·m). Such heat-transfer related effects are especially noteworthy when dealing with gas phase compounds. Based on these control experiments, we consider the monolayer-over-mirror to be the optimal configuration for our subsequent 3D SERS experiments.

### 3.3. Evaluation of the SERS Enhancement Factor and SERS Gain for 3D Fractals Active SERS Substrates

**Figure 8:**
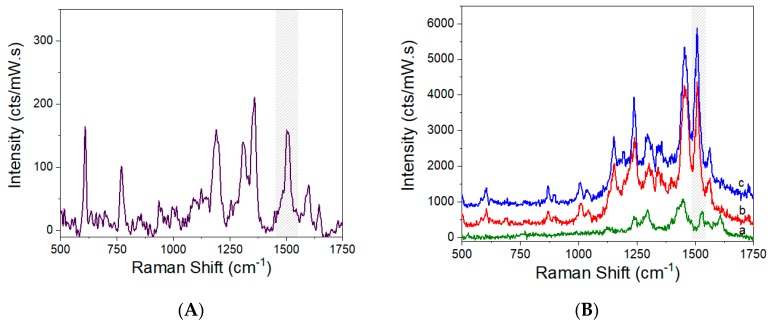
(**A**) Normal Raman spectrum (1000×) of aqueous solution R6G (1 mM). (**B**) SERS spectra of (1 µM) R6G on the SERS substrate: (a) Glass_Ag_AuNPs; (b) 1G_Ag_AuNPs; (c) 3G_ Ag_AuNPs.

Typical SERS spectra of R6G (1 mM) are presented in Figure 8 for the 2D and 3D SERS active substrates prepared in this work, as well as the normal Raman spectrum of R6G solution (1 mM) used as a reference for the calculation of EF-values (see Section 2.3). The corresponding EF-values, calculated following Equations (1)–(3), are summarized in Table 2.

The importance of the 3D-fractal architectures is demonstrated by comparing them with the planar Glass_Ag_AuNPs, where the latter gives rise to an enhancement factor of 1.9 × 10^4^, 6-fold and 18-fold lower than the 1G and 3G fractals covered with Ag_AuNPs, which have EF-values of 1.18 × 10^5^ and 3.51 × 10^5^, respectively. The enhanced surface area of 3D-fractals, due to their extension in z-direction, is already considered for the estimation of N_SERS_. Thus, the registered differences can only be attributed, due to the fractal size, to surface plasmon modes generated by the edges and corners, as observed previously for Sierspinki fractals with feature sizes of 750 nm, 1.2 µm and 4 µm [15].

**Figure 9:**
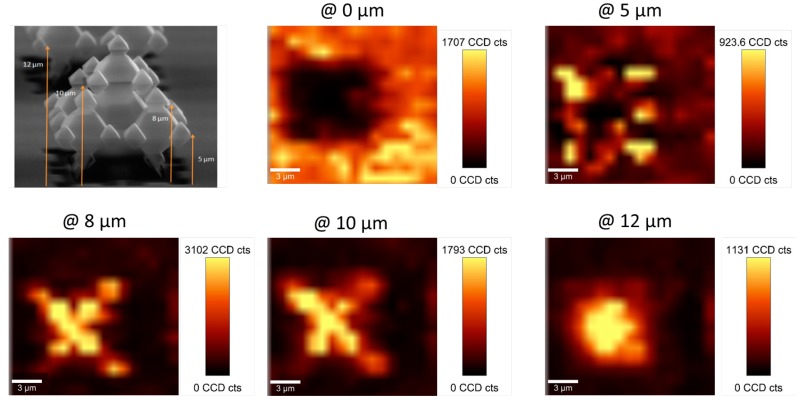
SERS Active 3G_Ag_AuNP substrates: SEM image and Raman signal mapping across a 20 × 20 µm at different heights (z-position) from the bottom: 0 µm (bottom surface); 5 µm; 8 µm; 10 µm; 12 µm. Conditions: 1 mW, 1 s. Recreation of the bright areas in a cross section view of the substrate.

To obtain an estimation of the EM enhancement on the fractal structures, the Raman signal was collected at selected z-positions in a mapping area of 20 × 20 µm (1 spectrum/µm), see Figure 9. Bright areas represent higher Raman intensity and comparing the xy-images at different z-positions, i.e., 5, 8, 10 and 12 µm from the ground level, it is evident the higher electromagnetic field on the corners, apices or tips of the microstructures. This enhancement is modest, just 1.7 folds higher, when compared with those exhibited by Sierspinki fractals with feature sizes of 150 nm [15]. However, due to the higher extension of the 3D fractal structures in the z direction, 12 µm vs. 40 nm, larger interfacial area for gas-solid contact and improved limit of detection for gas sensing could be expected.

### 3.4. SERS Detection in Gas Phase of Sarin Surrogate

**Figure 10:**
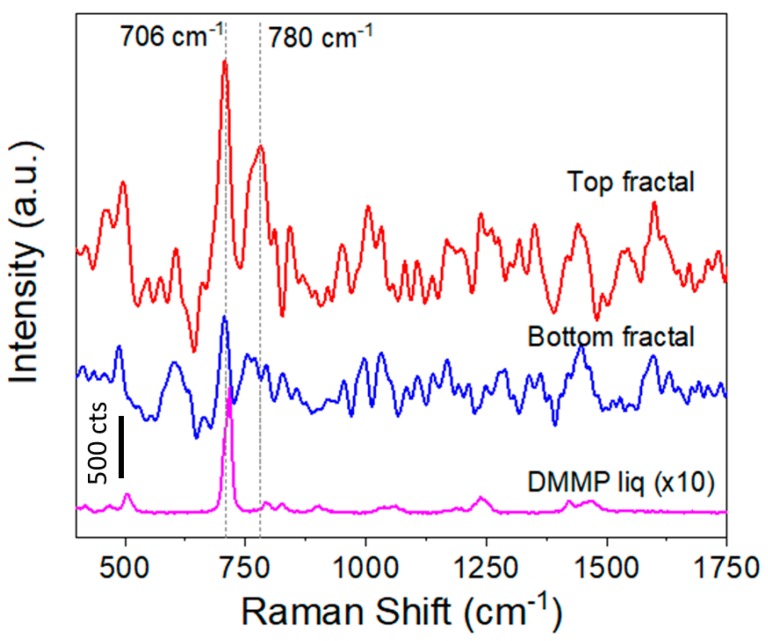
SERS spectra recorded on top and bottom of a 1G fractal sample (1G_Ag_AuNPs) upon exposure to 1.2 ppmV of DMMP in gas phase. A normal Raman spectrum of DMMP in liquid phase is included for comparison. Acquisition conditions: 5 mW and 1 s.

Figure 10 comparatively shows the recorded SERS spectra on a 1G_Ag_AuNPs sample upon exposure to DMMP in vapor phase (1.2 ppmV) with the focus plane at two z-positions: 0 µm (bottom part of the fractal) and 5 µm (top part of the fractal). The most intense signal is appearing at 706 cm^−1^ for both heights, tentatively assigned to the P-C stretching mode and used as Raman reference line. However, when focused at z = 0 the signal-to-noise ratio hinders its proper identification. In addition, the characteristic peak of DMMP at 780 cm^−1^, attributed to PO_2_ bending, is clearly observed on the top of the fractals. Both peaks are shifted with respect to normal Raman of the DMMP in liquid phase. This observation agrees with the hydrogen bonding type interactions between citrate from Au NPs and DMMP molecule, as described in the literature [21]. The citrate coating acts as an effective trap for the target molecules in the immediate vicinity of the metallic surface where the maximum electromagnetic enhancement is achieved. It is important to note that the spectra in Figure 10 are taken at 1s integration time, under these conditions a value of around 2000 cts is obtained, indicating a large enhancement of the electromagnetic field in the vicinity of the molecule. When the spectra were taken at longer acquisition times, the signal around 1600 cm^−1^ corresponding to amorphous carbon appeared, indicating that the high energy concentrated on the molecule was able to burn it. A similar result was obtained when 3G fractals were used for measurements. Thus, we can conclude that in the case of the DMMP molecule the energy should be modulated to avoid decomposition of the molecule and a trade-off between electromagnetic field enhancement and stability of the molecule should be achieved.

**Figure 11:**
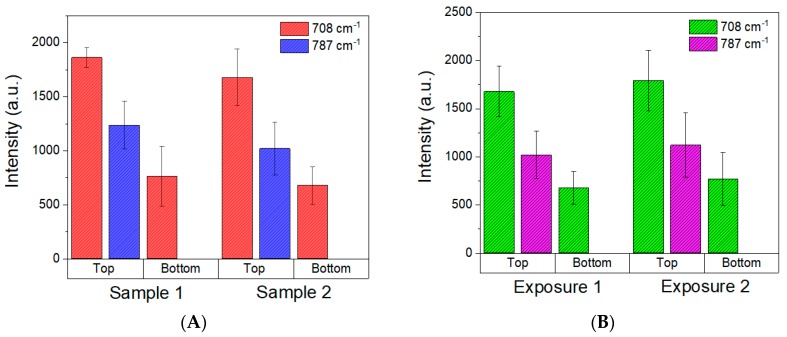
Column diagram represents the SERS intensity of the DMMP modes 708 and 787 cm^−1^ measured (**A**) on the different 1G_Ag_AuNP upon the exposure to DMMP (1.2 ppmV) and (**B**) on the same 1G_Ag_AuNP upon the double exposure to DMMP (1.2 ppmV). The SERS intensities are the average of 6 spots at different sample position, the error bars are the intensity standard deviation. Acquisition conditions: 5 mW and 1 s.

To evaluate the homogeneity of the SERS substrate and the reproducibility of the present fabrication method, two different 1G fractal samples were prepared with the same protocol, and 6 different spots were randomly selected on the top and on the bottom to record the SERS signal of 1.2 ppmV DMMP for each substrate (Figure 11A and Figure S2 of the Supplementary Materials). Sample 1 and 2 result in similar intensities with a relatively low standard deviation for the 6 spots measured, which is an evidence of the good reproducibility of the present fabrication method.

The SERS substrate reusability of the 1G_Ag_AuNP as SERS substrate was evaluated measuring the same substrate two times (Figure 11B). The 1G_Ag_AuNP was exposed to 1.2 ppmV of DMMP in gas phase (exposure 1 in Figure 11B), then it was purged with N_2_ for 25 min, until the DMMP fingerprint disappear. After 24 h, 1.2 ppmV of DMMP was fed again to the same SERS substrate (exposure 2). The measurements show a good repeatability in accordance to the reversible interactions between DMMP and Au@citrate NPs already described in our previous publication [24].

## 4. Conclusions

We have demonstrated the fabrication of efficient 3D-fractal active SERS substrates by combining corner lithography, anisotropic wet-etching of silicon and electrostatically driven Au@citrate NPs assembly. The fabrication procedure leads to hollow 3D fractals extended in z-direction up to 12 µm height that are coated with a homogeneous monolayer of Au@citrate NPs with a surface density above 600 NPs/µm^2^. The Au monolayer over Ag mirror configuration is preferred over bare 3D fractals, because then the SERS responses are less noisy and more reproducible. The SERS activity is demonstrated by means of real-time detection of DMMP, the commonly used surrogate of G-series nerve agents, at a low concentration of 1.2 ppmV. The hot spot densities extend along the *z*-axis with preferential accumulation on the corners and tips of the fractal microstructures. Further efforts are focused on SERS modelling by the Finite-difference time-domain (FDTD) method for optimization of the design of 3D-fractal structures with improved EM field enhancements.

## Figures and Tables

**Table 1:** Main characteristics of the SERS substrates and reference samples studied in this work.

Substrates	Sample	Density Au@citrate NPs (AuNP/µm^2^)
References	Glass_Ag	-
	Glass_AuNPs	623 ± 8
2D active SERS	Glass_Ag_AuNPs	633 ± 30
3D-fractal active SERS	1G_Ag_AuNPs	619 ± 4
	3G_Ag_AuNPs	561 ± 16

**Table 2:** Enhancement factor and SERS gain for the 2D and 3D SERS substrates studied in this work.

R6G Concentration	Parameter	Glass Ag_AuNP	1G Fractal Ag_AuNP	3G Fractal Ag_AuNP
1 mM, liquid droplet	I_Raman_ (cts/mW·s)	0.148	0.148	0.148
	N_Raman_ (molecules)	1.25 × 10^6^	1.25 × 10^6^	1.25 × 10^6^
1 µM, 2 µL dried droplet	I_SERS_ (cts/mW·s)	410	4038	4975
	N_SERS_ (molecules)	1.92 × 10^6^	2.9 × 10^6^	1.2 × 10^6^
	EF	1.9 × 10^4^	1.18 × 10^5^	3.51 × 10^5^
	SERS Gain	2.91 × 10^7^	2.73 × 10^8^	3.36 × 10^8^

## Supplementary Materials

The following are available online at http://www.mdpi.com/2072-666X/9/2/60/s1. Figure S1: UV-VIS spectra of the SERS substrates herein studied: Glass_ Ag, Glass_AuNPs, Glass_Ag_AuNPs, 1G_Ag_AuNPs, 3G_Ag_AuNPs; Figure S2: SERS spectra of 6 spots recorded on bottom and top of 1G_Ag_AuNPs sample upon exposure to 1.2 ppmV DMMP in gas phase.
