# Supplementary material for: 3D Fractals as SERS Active Platforms: Preparation and Evaluation for Gas Phase Detection of G-Nerve Agents
Source: Micromachines (Basel). 2018 Jan 31;9(2):60. doi: 10.3390/mi9020060 (PMC6187359; doi:10.3390/mi9020060)
Supplement: Supplementary file 1 [file micromachines-09-00060-s001.pdf]

3D Fractals as SERS Active Platforms: Preparation and Evaluation for Gas Phase Detection of G-nerve Agents

Marta Lafuente, Erwin J. W. Berenschot, RoaldM Tiggelaar, Reyes Mallada, Niels R. Tas *, Maria P. Pina *

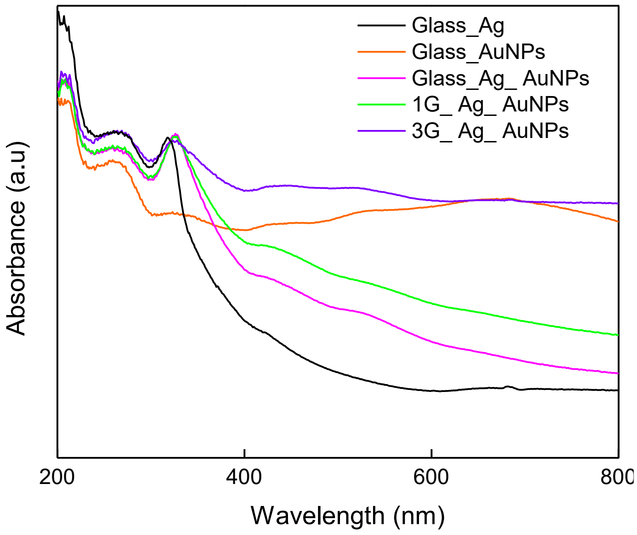

Figure S1. UV-VIS spectra of the SERS substrates herein studied: Glass_ Ag, Glass_AuNPs, Glass_Ag_AuNPs, 1G_Ag_AuNPs, 3G_Ag_AuNPs.

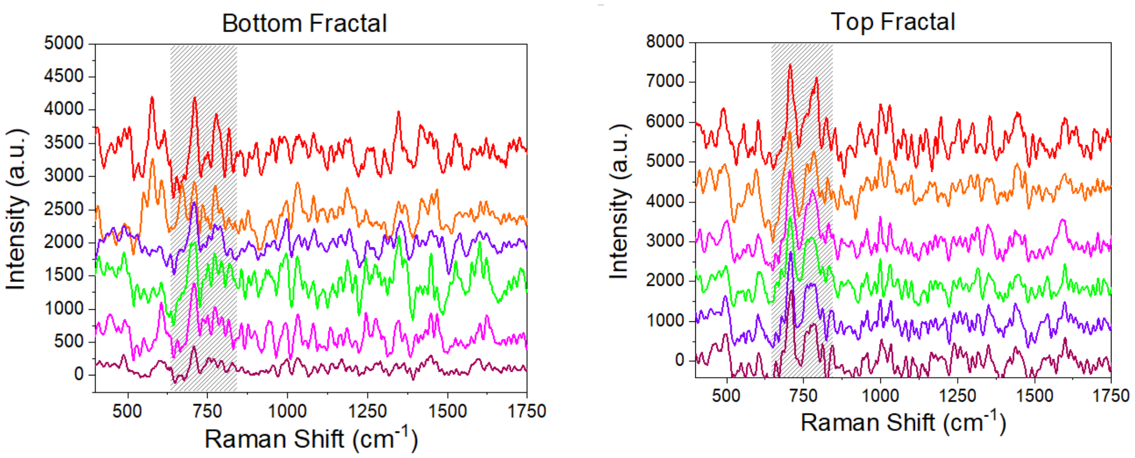

Figure S2. SERS spectra of 6 spots recorded on bottom (**left**) and top (**right**) of 1G_Ag_AuNPs sample upon exposure to 1.2 ppmV DMMP in gas phase.
